# Neuropathic Pain in Multiple Sclerosis and Its Animal Models: Focus on Mechanisms, Knowledge Gaps and Future Directions

**DOI:** 10.3389/fneur.2021.793745

**Published:** 2021-12-16

**Authors:** Ersilia Mirabelli, Stella Elkabes

**Affiliations:** ^1^Reynolds Family Spine Laboratory, Department of Neurosurgery, New Jersey Medical School, Rutgers the State University of New Jersey, Newark, NJ, United States; ^2^Department of Biology and Chemistry, School of Health Sciences, Liberty University, Lynchburg, VA, United States

**Keywords:** experimental autoimmune encephalomyelitis (EAE), glia and neuroinflammation, pain, ion channel, ion pumps, ion exchangers/transporters

## Abstract

Multiple sclerosis (MS) is a multifaceted, complex and chronic neurological disease that leads to motor, sensory and cognitive deficits. MS symptoms are unpredictable and exceedingly variable. Pain is a frequent symptom of MS and manifests as nociceptive or neuropathic pain, even at early disease stages. Neuropathic pain is one of the most debilitating symptoms that reduces quality of life and interferes with daily activities, particularly because conventional pharmacotherapies do not adequately alleviate neuropathic pain. Despite advances, the mechanisms underlying neuropathic pain in MS remain elusive. The majority of the studies investigating the pathophysiology of MS-associated neuropathic pain have been performed in animal models that replicate some of the clinical and neuropathological features of MS. Experimental autoimmune encephalomyelitis (EAE) is one of the best-characterized and most commonly used animal models of MS. As in the case of individuals with MS, rodents affected by EAE manifest increased sensitivity to pain which can be assessed by well-established assays. Investigations on EAE provided valuable insights into the pathophysiology of neuropathic pain. Nevertheless, additional investigations are warranted to better understand the events that lead to the onset and maintenance of neuropathic pain in order to identify targets that can facilitate the development of more effective therapeutic interventions. The goal of the present review is to provide an overview of several mechanisms implicated in neuropathic pain in EAE by summarizing published reports. We discuss current knowledge gaps and future research directions, especially based on information obtained by use of other animal models of neuropathic pain such as nerve injury.

## Introduction

Multiple sclerosis (MS) is the most common chronic inflammatory and demyelinating disease of the central nervous system (CNS) (1, 2). MS is a multifaceted disease. Both genetic and environmental factors contribute to the risk of developing the disorder. It is estimated that close to a million people live with MS in the United States alone (3). Young adults are the most affected with the range of onset being 20–40 years of age. The prevalence of the disease is three-times higher in women than in men (4, 5). Even if the majority of the patients initially present with relapsing-remitting (R-R) MS, the disease eventually progresses into secondary-progressive form within 10–20 years from the onset (6). The major symptoms include limb weakness, fatigue, spasticity, sensory impairments, loss of coordination, cognitive decline, pain and paralysis. Because MS is not curable, the goal of the various therapeutic approaches is to slow disease progression and alleviate the symptoms to improve quality of life (7).

Among the MS population, pain is a frequent symptom that affects from 28 to 87% of individuals, with variations in time of onset and type of pain. Pain impacts both the physical and emotional well-being of the individual (8) and interferes with the majority of daily life activities such as sleep, work, and participation in recreational and social activities (9), reducing the quality of life and leading to depression and other comorbidities (10–12).

The pain associated with MS can be classified into four groups: musculoskeletal pain (painful tonic spasms, pain secondary to spasticity), intermittent central neuropathic pain (trigeminal neuralgia, Lhermitte's sign), continuous central neuropathic pain, and mixed neuropathic and non-neuropathic pain (headache). Regardless from the type of pain, there is a correlation between pain and the disease course, its duration, and age of the affected individual (13). Neuropathic pain is more common in women with higher disability and longer disease duration (14).

Painful spasms, especially in the lower limbs, are due to ectopic impulses, generated from the motor fibers, as a result of axonal damage and demyelination. These painful spasms are more frequent at night (15). Headaches and low back pain are very common among affected individuals throughout the course of the disease (16–18). Importantly, other manifestations of pain occur as the disease progresses. The spasticity and progressive weakness compromise the posture and motility of the individual, leading to osteoporosis and the dysfunctions of tendons, ligaments and/or joints, which evoke secondary pain (15). Even the pharmaceutical treatments commonly used for MS symptomatology exacerbate some of the most common pains (19). For example, Interferon-β exacerbates headaches and migraines (20).

### Pain Circuits and Mechanisms Underlying Peripheral and Central Sensitization

Primary sensory neurons of the dorsal root ganglia (DRG) sense noxious stimuli via their peripheral projections, and convey the pain information to the dorsal horn (DH) of the spinal cord (SC) through their central projections. The central projections synapse with second-order sensory neurons and excitatory or inhibitory interneurons in the DH. The DH also receives projections from supraspinal locations which modulate pain transmission. These signals are integrated in the DH and then conveyed to various brain regions where pain perception and affective responses to pain develop (21). The cingulate and insular cortices, the amygdala and brainstem, are among brain regions implicated in pain states (22–25).

Neuropathic pain is caused by damage or disease that affects the central or peripheral somatosensory systems, and is referred to as central or peripheral neuropathic pain, respectively (26, 27). The pathophysiological changes associated with neuropathic pain include hyperexcitability of neurons in pain pathways. Neuronal hyperexcitability is an essential mechanism underlying the increased sensitivity to pain in various pathological conditions.

Central sensitization manifests as increased sensitivity to innocuous (allodynia) or noxious (hyperalgesia) stimuli, or spontaneous pain. It occurs in many chronic pain conditions (28–30) including MS or its animal models (31) and is independent of peripheral damage or disease. Central sensitization is the consequence of maladaptive changes in pain circuits of the CNS and heightened excitability of neurons which is partly attributed to increased synaptic efficacy and reduced inhibition (32). The major mechanisms underlying central sensitization have been discussed in earlier comprehensive reports (33, 34) and include glutamate and glutamate receptors. The efficacy of excitatory synapses is enhanced, partly as a consequence of increased glutamate release, impaired glutamate uptake and overactivation of glutamate receptors in DH neurons involved in pain processing. Increased activity, expression and trafficking of ionotropic glutamate receptors, such as α-amino-3-hydroxy-5-methyl-4-isoxazolepropionic acid (AMPA) and N-methyl-D-aspartate (NMDA) receptors are observed in second order sensory neurons of the DH (34–36). Metabotropic glutamate receptors (mGluRs) also participate in the modulation of pain transmission by inducing Ca^2+^ release from intracellular stores, which, in turn, activates kinases, including phosphatidyl inositol 3 kinase (PI3K) and mitogen activated protein kinase (MAPK). Activated kinases phosphorylate ion channels and receptors implicated in pain mechanisms, altering their activity and leading to increased synaptic efficacy (33). These events, together with decreased inhibitory activity of gamma aminobutyric acid (GABA)ergic interneurons (34) and the additional effects of non-neuronal cells, enhance neuronal excitability. In particular, activated glia and infiltrating immune cells secrete pro-nociceptive cytokines, including Tumor Necrosis Factor α (TNFα) and Interleukin 1β (IL-1β), which increase the excitatory and reduce the inhibitory currents in DH neurons, supporting central sensitization (37). Reactive astrocytes have been associated with hyperalgesia under pathological conditions (38). Furthermore, descending noradrenergic and serotoninergic projections to the DH inhibit or facilitate pain transmission (23). Therefore, injury- or disease- induced aberrance in descending pain pathways modulate chronic pain (39). For example, in individuals with MS, lesions in the periaqueductal gray (PAG) are often reported (40). The PAG is an important control center for the modulation and propagation of pain along the descending pathways with analgesic effect upon stimulation (40). In contrast, the rostral ventromedial medulla (RVM) GABAergic cells project to the DH where they facilitate mechanical nociception by inhibiting the spinal GABAergic interneurons (41). Finally it is important to mention that supraspinal glia also contribute to the modulation of chronic pain along the descending pathways particularly through the release of soluble mediators (42).

Peripheral sensitization manifests as an intensification of the responsiveness of primary sensory neurons in the DRG and lowered pain thresholds when there is damage or pathology in tissues that they innervate. Stimulus-independent spontaneous pain is also one of the outcomes of peripheral sensitization. Multiple effectors, including chemokines, prostaglandins, calcitonin gene-related peptide (CGRP), adenosine triphosphate (ATP), substance P and nerve growth factor (NGF) are released at the affected site (43–46). These effectors initiate a molecular cascade partly mediated by tyrosine kinase- and G-protein-coupled receptors. Protein kinases are activated and phosphorylate ion channels and receptors at the peripheral terminals of nociceptors, and by doing so, alter their activity (47–49). In addition, the same modulators modify the expression, localization and stability of nociceptive ion channels and receptors (50). The overall outcomes of these alterations are increased activity of nociceptors and decreased pain thresholds (51).

### Manifestation of Neuropathic Pain in MS

Neuropathic pain is widely experienced among individuals with MS and can take several forms. Dysaesthetic (lower extremity) pain is the most common form of neuropathic pain described as a constant burning, tingling and throbbing, painful sensation in the legs and feet (10). Even though the specific mechanisms underlying the onset of dysaesthetic pain have not been elucidated, it has been suggested that lesions in the SC could lead to the disruption of pain transmission along the spinothalamic tract, or dysfunction of GABAergic interneurons (52). In accordance, magnetic resonance imaging (MRI) of individuals with dysaethetic extremity pain shows plaques in the cervical and thoracic spinal cord (53, 54).

Trigeminal neuralgia is described as a brief electric shock sensation resulting from the irritation of the trigeminal nerve. It primarily affects different facial regions. Pain can be induced even by mild stimulation of the face. Although the attacks can be brief (up to 2 min), they occur frequently during the day (up to 50/day) (55). Demyelination of the pons, which affects the trigeminal root entry zone, and neurovascular compression of the trigeminal root are observed in MS, and could potentially be a cause of trigeminal neuralgia (55). Similar to trigeminal neuralgia, Lhermitte's sign is described as an electric-shock sensation associated with neck movement, and running along the back and down the limbs (56). Lhermitte's sign is not specific to MS and manifests in other pathological conditions that include the compression or lesion of the cervical SC (57). In accordance with this idea, the MRI of individuals with MS shows demyelinated plaques in the dorsal columns at the cervical level (58).

### Treatment of Neuropathic Pain in MS

The treatment of neuropathic pain is challenging, mostly due to the low efficacy and side effects of pharmacological agents (59–61). Tricyclic antidepressants, and serotonin/norepinephrine reuptake inhibitors have been used as first-line pharmacological treatments. Anticonvulsants, including gabapentine and pregabalin, are also considered first-line of treatment (62). Although cannabinoids and opioids have been utilized for the alleviation of neuropathic pain in MS, they are not considered as a first-line treatment option (63, 64). Neuromodulation therapies are becoming more common in clinical practice (65). Both brain and SC chemical and/or electrical stimulation and inhibition have been used for the management of different chronic pain conditions (66). Transcranial Direct Current Stimulation (67), peripheral nerve field stimulation (68), transcutaneous spinal direct current stimulation (69), are among treatments to manage neuropathic pain in MS. Alternative strategies such as water exercise and yoga have also been utilized (70).

The goal of the present review is to highlight select mechanisms implicated in the development and persistence of chronic neuropathic pain in MS and its animal models. We first focus on the cellular mechanisms, and then discuss potential molecular mechanisms with particular emphasis on ion channels, pumps and exchangers.

## Animal Models of MS: Experimental Autoimmune Encephalomyelitis (EAE)

Experimental Autoimmune Encephalomyelitis (EAE) is one of the best characterized animal models utilized for the study of MS (71–73). For over 30 years, the use of EAE in different susceptible animal strains has proven essential for investigations on various aspects of MS pathophysiology, and the discovery of drugs that are widely used to treat MS such as Interferon-β or Glatiramer acetate (74–76). With all the limitations that an animal model could present, conventional EAE models and the spontaneous EAE model observed in T-cell receptor transgenic mice (77), have shed light on many aspects of MS etiology, pathophysiology and its pharmacological treatments (72, 78–80).

### Induction of EAE

EAE can be induced by immunization of susceptible animal strains with myelin components and encephalitogenic peptides, or by adaptive transfer of encephalitogenic T cells (81). There are various EAE models in different species and susceptible strains, and they mimic distinct hallmarks of MS (82). In particular, a clear distinction can be made between two forms of EAE: chronic-progressive and R-R EAE. To induce chronic-progressive EAE, mice are inoculated with myelin oligodendrocyte glycoprotein (MOG). The MOG antigen in CNS tissue homogenates was long recognized to be crucial for provoking demyelinating lesions characteristic of MS (83). Currently, the use of recombinant MOG or synthetic encephalitogenic fragments such as MOG_35−55_ for immunization provides a reproducible animal model for the study of MS pathology (73). Alongside with MOG, myelin basic protein (MBP) has been used to induce chronic-progressive EAE (84). Proteolipid protein (PLP) and its encephalitogenic PLP fragment (PLP_139−151_) have been used as a standard approach to induce R-R EAE in susceptible rodent strains. The recombinant protein or synthetic peptide of choice is dissolved in a mineral oil-based adjuvant, Complete Freund's adjuvant (CFA), containing heat-inactivated mycobacteria tuberculosis (MT) that activates the innate immune system through actions on pattern recognition receptors. Injections of pertussis toxin enhance the immune response and perturb blood brain barrier integrity. The onset, course and severity of EAE differ depending on the antigen and adjuvant utilized, and the species, strain, sex and age of the animals used. Within the same species and strain, susceptibility to EAE can be different based on intrinsic and environmental factors such as colonization of the gut and the type of commensal flora, elements that are challenging to control (85).

Alongside with the classical EAE forms, induced by sensitization to a specific myelin protein, there are spontaneous and humanized EAE models which have been discussed in a comprehensive review (86). Among those models are two that have been developed by use of transgenic mouse lines: opticospinal EAE and spontaneous R-R EAE. These models have been useful to unravel the role of B cells and B and T cell interactions in disease pathogenesis (87). Mice manifesting opticospinal EAE develop a chronic progressive EAE-like disease that affects primarily the optic nerve and the SC, but not the brain. The neurological deficits that spontaneously develop are predominantly reminiscent of neuromyelitis optica (88). However, transcriptome profiling indicated that human MS risk genes are among differentially regulated transcripts, supporting the applicability of this model to the study of MS etiology (89). Mice affected by spontaneous R-R EAE develop the disease at high frequency and manifest unique clinical features with distinct symptoms at onset (e.g., ataxia) and during relapse (e.g., hindlimb paralysis). This is especially observed in female mice. The clinical symptoms are paralleled by formation of lesions in relevant CNS regions (e.g., lesions in the cerebellum and brainstem in mice manifesting ataxia, and SC lesions in mice with hindlimb paralysis) (90). Therefore, the spontaneous R-R EAE is a useful animal model for the study of specific disease aspects and treatments because it mimics the most frequent form of MS (88, 90).

Neuroinflammation, demyelinating lesions, axonal damage, and oligodendrocyte and neuronal death are observed in classical EAE, with the SC being the CNS region most affected. These histopathological features are similar to MS neuropathology (91, 92).

### EAE Symptoms

Ascending paralysis is the most pronounced symptom in animals with classical EAE. It manifests several days post-immunization, and is associated with disease progression (93). In chronic-progressive EAE, flaccid paralysis starts at the tail with loss of tone, proceeds to the hind and fore limbs, and could lead to death. Aside locomotor impairment, sensory and cognitive dysfunctions are also observed even before the manifestation of motor deficits (94, 95). This is in concordance with the impairment in cognitive function in MS, which is observed even at early phases of the disease, and is paralleled by demyelination and neuronal damage that affects the gray matter (96, 97).

As in the case of MS, rodents with EAE manifest pain (98–100). Pain behavior has been assessed in both chronic progressive and R-R EAE (98–100). Mechanical and thermal allodynia have been documented at onset of EAE symptoms, and concomitant with deficits in cognitive behavior (101), which has also been reported in MS patients (102). Similar to MS, pain behavior and hypersensitivity are variable among different EAE models, revealing the heterogeneity of the disease and mimicking the diversity among individuals with MS (103). Neuroinflammation and disease severity modulate mechanical hypersensitivity, in a MOG_35−55_-induced EAE model (104). Similar to the caudal to rostral progression of the neuroinflammatory reaction observed in the SC, ascending sensitization might manifest during EAE. Both morphological and functional changes influence the cortical synapses participating in central sensitization and pain hypersensitivity during EAE (105). An increase in immune cell infiltration, glial activation and release of soluble inflammatory mediators (106) together with alterations in glutamate and GABA levels as well as changes in neurotransmission lead to an overall increase in neuronal excitability in EAE (107, 108).

Because anxiety and depression are strongly associated with pain in individuals with MS, EAE has been widely used for the study of these comorbidities (109–111). Mice, affected by a mild form of EAE that does not substantially affect motor function, were evaluated at a later disease stage. Increased anxiety- and depression-like behavior correlated with elevated TNFα, neuronal dysfunction, synapse loss, and neuroinflammation-induced hippocampal damage (112).

### Cellular and Molecular Mechanisms Underlying CNS Damage in EAE

EAE has been useful to unravel several cellular and molecular mechanism implicated in MS pathogenesis including the involvement of T cells and other infiltrating immune cells, and the role played by humoral components of the immune system in neurodegeneration. In fact, during the initial phase of EAE, T cells play the major role. Upon their activation in the periphery, T cells produce and secrete pro-inflammatory cytokines and cross the blood-brain barrier. The entry of T cells into the CNS is mediated by integrins and cell adhesion molecules which are upregulated. Once in the CNS, these T cells are re-activated by antigen presenting cells (113). The presentation of myelin-derived antigens by macrophages to T cells, the infiltration of additional immune cells including macrophages and B cells, and glial activation lead to a complex and extensive neuroinflammatory reaction, ultimately resulting in demyelination, loss of oligodendrocytes and neurons, and axonal degeneration (72). The cytotoxic effects of cytokines, the activation of complement proteins and the production and secretion of reactive oxygen and nitrogen species (ROS and RNS, respectively) are among molecular mechanisms that cause CNS damage (114). The free radicals have a severe and deleterious impact on neuronal metabolism, mitochondrial integrity, and energy balance leading to increased intracellular calcium (Ca^2+^), and eventually neuronal death (115). Because of the important role played by oxidative stress in MS, some of the latest therapies focus on reducing the deleterious effects that are the result of ROS and RNS accumulation (116). Oxidative stress does not only damage neurons, but it affects glial cells as well, with oligodendrocytes being the most sensitive cell type (117). In active lesions during the early stages of R-R MS, oxidative damage affects primarily oligodendrocytes and myelin (117, 118). The accumulation of ROS or RNS in mitochondria compromises oligodendrocyte function and impairs the differentiation of oligodendrocyte progenitor cells. This could be a mechanism underlying the impairment of remyelination (119) since mitochondria are essential for the biosynthesis of lipids to produce myelin (120, 121). Oxidative stress affects oligodendrocytes and their precursors in more than one way and therefore, could contribute directly and indirectly to MS pathophysiology (122, 123).

## Role of Activated Glia and Glia-Neuron Interactions in Neuropathic Pain

Following injury or disease, glia undergo morphological and functional changes switching into an active state. As indicated above, glial activation also occurs in MS and EAE, and is considered an essential determinant of disease pathology (124). In addition to myelin phagocytosis and antigen presentation, glia release pro-inflammatory and pro-nociceptive cytokines, chemokines, brain derived neurotrophic factor (BDNF), ROS, and ATP, leading to both neurotoxicity (125) and the development and maintenance of neuropathic pain (126–131). Attenuation of astrocyte and microglia activation using fingolimod has been associated with amelioration of pain hypersensitivity in mice with EAE (31).

Activated glia are also a source of glutamate. Glutamate homeostasis is impaired in MS and EAE. Glutamate levels in the cerebrospinal fluid (CSF) of individuals with MS correlate with disease severity (132). Increased serum glutamate levels and elevated glutamate in active white matter lesions have been observed in MS (133, 134). Since glutamate and glutamate receptors play essential roles in the hyperexcitability of sensory neurons and sensitization to pain, increased glutamate release by glia potentially contributes to neuropathic pain in MS/EAE. Glutamate can also induce excitotoxicity through the activation of ionotropic receptors which participate in the neurodegeneration observed in MS and EAE and other CNS disorders (135, 136). In addition to neurons, glia express ionotropic and metabotropic glutamate receptors, and therefore, their function could be modulated via direct stimulation by glutamate (137, 138).

Astrocytes are essential for the clearance of synaptic glutamate through uptake by the glutamate transporters, glutamate aspartate transporter (GLAST/EAAT1) and glutamate transporter 1 (GLT-1/EAAT2). A decrease in EAAT1 and EAAT2 levels was observed in the SC at the peak of EAE which persisted even after remission (139). A reduction of astrocyte-mediated glutamate transport could elevate glutamate levels causing neuronal hyperexcitability as well as excitotoxicity. Oligodendrocytes also express EAAT1 and EAAT2 and aberrance in glutamate uptake by oligodendrocytes also contributes to pathology in EAE/MS (140). Paradoxically, an increase in glial glutamate transporter levels in the MS optic nerve has been implicated in neuroprotection (141). A loss of EAAT1 and EAAT2 in MS lesions and an upregulation of EAAT2 in the adjacent cortex with intact myelin has been documented (142). Since both astrocytes and the white matter oligodendrocytes express glutamate transporters (143), these contradictory findings could be the result of many factors including cell type, CNS region, disease stage and whether EAE as opposed to post-mortem MS tissue were analyzed. Collectively, the studies support the notion that glial EAAT1 and EAAT2 could play a role in EAE/MS pathology. However, their precise contribution to neuropathic pain in these diseases requires further investigations.

Glutamate receptor antagonists have been assessed in EAE, primarily in the context of neuroprotection, prevention of demyelination, and improvement of motor deficits (144–148). Although glutamate receptor antagonists, including memantine (149–151) or modulators of the glutamatergic system such as ketamine (152, 153) have been used for the relief of chronic pain in various diseases or injuries, their effectiveness in the alleviation of neuropathic pain during EAE/MS has not been adequately investigated.

## The Role of Ion Channels, Pumps and Exchangers in Neuropathic Pain: Evaluating the Potential Contribution to Pain in EAE

### Ion Channels

Ca^2+^, Na^+^, and K^+^ channels are essential components of pathways underlying pain mechanisms (154, 155). They have also been implicated in several aspects of MS pathophysiology (156). Voltage gated Na^+^ and Ca^2+^ channels (VGSCs and VGCCs, respectively) are expressed in sensory neurons of the DRG and in DH neurons (157, 158). Under physiological conditions, these channels participate in neuronal excitability and neurotransmitter release (159, 160). Following CNS and peripheral nervous system (PNS) injury and disease, changes that affect their expression and distribution lead to neuronal hyperexcitability, resulting in an exaggerated and repetitive response to subthreshold sensory stimuli, and increasing synaptic strength (161, 162). The increased activity of Na^+^ and Ca^2+^ channels in first order sensory neurons of the DRG promotes neurotransmitter release, which, in turn, enhances the excitatory inputs to the SC (34).

### CaV2.2

The N-type VGCC, CaV2.2, is one of the major ion channels regulating neurotransmitter release in the PNS and CNS (163). Compelling evidence indicates that CaV2.2 is involved in pain mechanisms. In fact, CaV2.2 has been extensively investigated, especially with the goal of finding pharmaceutical approaches for the treatment of chronic pain (164–166).

Several lines of evidence support the involvement of CaV2.2 in neuropathic pain.

Following CFA-induced inflammatory pain in mice, CaV2.2 mRNA and protein levels are upregulated in the lumbar DRG and correlate with the increase in thermal hyperalgesia (167). Electrophysiological recordings from the DRG showed a voltage increase in CaV2.2 currents and a rise in the frequency of spontaneous action potentials that were directly related to CaV2.2. These factors, taken together, suggested an overall enhancement of the excitability of DRG neurons (167).

Furthermore, CaV2.2 knockout mice manifest decreased pain responses in models of neuropathic and inflammatory pain (168). Blockade of CaV2.2 in nociceptors reduces chronic inflammatory pain in a mouse model of rheumatoid arthritis (169). Additionally, interference with CaV2.2 trafficking to the membrane of DRG neurons abrogates Ca^2+^ currents, decreases stimulus-induced pro-nociceptive neuropeptide release, and reduces the excitatory synaptic transmission in lamina II DH neurons which receive DRG afferents that express CaV2.2. These changes result in the attenuation of pain responses in several rodent models of evoked inflammatory and neuropathic pain (170). Following chronic constriction injury (CCI) of the sciatic nerve, CaV2.2 is upregulated in lamina II of the lumbar DH (171). In the lumbar L5-spinal nerve ligation (L5-SNL) model, IL-1β and IL-10, pro- and anti-nociceptive cytokines, respectively, modulate CaV2.2 expression in the DRG in opposite manner. Injury at the L5 level increases IL-1β release in the adjacent, uninjured L4 DRG which upregulates CaV2.2, resulting in neuronal hyperexcitability (172) and mechanical allodynia (173). In contrast, L5-SNL upregulates both IL-1β and IL-10 in the corresponding, injured L5 DRG and lumbar SC. However, the effects of IL-10 predominate and a significant reduction in CaV2.2 is observed (172). Collectively, the aforementioned studies illustrate the role played by CaV2.2 in pain.

Various mechanisms have been implicated in CaV2.2-mediated pain responses.

In peripheral somatosensory neurons, CaV2.2 is located close to synaptic vesicles containing pro-nociceptive neurotransmitters such as glutamate, substance P and CGRP (174). Inhibition of N-type VGCC in rat DRG neuronal cultures decreases the entry of Ca^2+^ and reduces neurotransmitter release (175). As indicated above (170), by regulating the release of pro-nociceptive neurotransmitters, CaV2.2 can enhance pain responses.

CaV2.2 forms a complex with NMDA receptors (NMDA-R) in the mouse and human SC (176). Following nerve injury, CaV2.2 promotes the trafficking and synaptic targeting of NMDA-R, suggesting an additional mechanism by which it could contribute to pain signaling (176).

Ectopic fiber sprouting in targets of DRG neurons, which occurs following nerve injury, has been associated with neuropathic pain (177). Inhibition of CaV2.2 activity in neurons derived from the DRG of mice manifesting CFA-induced inflammatory pain, prevented neurite outgrowth. It has been proposed that excess neurite outgrowth causes an increase in the nerve terminal density, which leads to increased nociceptive inputs and pain (167, 178).

During MS and EAE, a CaV2.2 subunit is overexpressed in active MS lesions, and MS/EAE plaques (179). It has been suggested that the overexpression of CaV2.2 impacts neuronal function and demyelination in the lesions (180, 181). Although a direct study has not yet confirmed the link between CaV2.2 and neuropathic pain in MS/EAE, the involvement of this channel in EAE pathophysiology, its role in neuropathic pain, together with the use of N-type Ca^2+^ channel blockers in the treatment of chronic pain in MS patients (182–184), point toward the need for further investigations to determine how CaV2.2 plays a role in EAE-associated pain.

### Nav1.6 and Nav1.8

Nav1.6 and Nav1.8 are among the VGSCs participating in inflammation and neuronal pathology in retinal ganglion cells and lumbar DRG during EAE (185–188). Nav1.6 is associated with Na^+^/Ca^2+^ exchangers (NCX). It has been proposed that in EAE, increased Nav1.6 expression prevents activity reverses the Na^+^/Ca^2+^ exchanger activity, and by doing so, promotes Ca^2+^ influx into neurons and causes neurodegeneration. It is possible to speculate that such interactions between Nav1.6 and the NCX also occur in sensory neurons and leads to elevated intracellular Ca^2+^ which, in turn, modulates pain mechanisms. Despite studies showing the role of Nav1.6 in various pain models, illustrated by examples described below, its contribution to MS- or EAE-associated pain has been underappreciated.

In neuropathic pain induced by SNL, siRNA-mediated Nav1.6 knockdown decreases the hyperexcitability of DRG neurons by reducing the frequency of the action potentials and restoring their resting potential. In addition, a decrease in sympathetic sprouting and the ectopic firing of Aβ fibers in the lumbar DRG have been documented (189). Moreover, in spared nerve injury, a model of neuropathic pain, conditional adenoviral vector (AVV)-mediated Nav1.6 knockdown in DRG neurons reduces excitability and ameliorates pain in adult mice. Furthermore, the accumulation of Nav1.6 in newly formed nodes of Ranvier, which has been implicated in pain, is mitigated by Nav1.6 knockdown (190). Accordingly, an amelioration of pain sensitivity is observed. Nav1.6 is also upregulated in a mouse model of diabetes and diabetic neuropathy (191). A gain of function mutation in Nav1.6 was found in individuals with trigeminal neuralgia. The mutated form shows decreased current threshold, increased frequency of evoked action potentials and overall potentiation of the Na^+^ currents with higher excitability of the trigeminal ganglion (192). Taken together, the investigations mentioned above stress the pivotal role played by Nav1.6 in pain states. The role of Nav1.6 could be a promising research direction to further explore in MS- and EAE-associated pain.

In the context of MS and EAE, NaV1.8 was first associated with alterations in the firing pattern of cerebellar Purkinje cells, causing dysfunction and disruption of motor coordination, one of the symptoms observed in EAE and MS (188). Few studies have shed light into its involvement in DRG sensitization. There is a significant increase in Nav1.8 levels in DRG neurons in CFA-induced inflammatory pain. TNFα and IL-1β directly modulate Nav1.8-like currents by activating p38/MAPK signaling pathway. This is paralleled by increased excitability of DRG neurons (160). Nav1.8 channels are expressed in more than 90% of the nociceptive neurons in the DRG (193). Since medium to large sensory neurons of the DRG are hyperexcitable at onset of EAE, and IL-1β is significantly elevated in the DRG at the same stage of the disease (194), it is possible that similar mechanisms contribute to peripheral sensitization and neuropathic pain associated with EAE. This possibility requires further investigations.

### K^+^ Channels

The calcium-sensitive large conductance K^+^ channels (BK) have recently been implicated in pain mechanisms during EAE (195). These channels are found in DRG neurons of mice with EAE and play a role in the endoplasmic reticulum (ER) stress-mediated pain response. It has been hypothesized that the prolonged neuroinflammation that occurs in the CNS during MS/EAE affects DRG neurons by conveying retrograde stress signals, which, in turn, induce ER stress (195). In fact, ER stress markers are elevated in post-mortem DRG of individuals with MS. Furthermore, 4-phenyl butyric acid (4-BPA), an inhibitor of ER stress, suppresses mechanical hypersensitivity in mice with EAE (195). The potential mechanisms by which ER stress leads to pain states in EAE have been investigated. ER stress causes variations in intracellular Ca^2+^ transients. Under physiological conditions, because of their Ca^2+^ sensitivity, BK channels respond to transient changes in Ca^2+^ concentrations by mediating the efflux of K^+^ in order to maintain the membrane potential of excitable cells. In mice affected by EAE and individuals with MS, there is a reduction in the b4 auxiliary subunit of the BK channels in DRG neurons (195). This subunit modulates the Ca^2+^ sensitivity of BK channels, and therefore, its electrical properties (196). The decrease in b4 subunit causes a change in BK channel physiology resulting in a higher depolarizing resting potential due to decreased BK activity and the release of K^+^ from neurons. An elevated depolarizing resting potential results in frequent and easier firing, ectopic discharges, and increased neurotransmitter release leading to hyperexcitability of sensory neurons. In mice with EAE, administration of 4-BPA restores the membrane potential of DRG neurons to physiological values by increasing the expression of b4 and by restoring BK channel properties.

A link between ER stress and pain behavior has been observed in other models of neuropathic pain. An increase in ER stress is recorded in the DH of rats following SNL and it corresponds with pain hypersensitivity. Inhibition of ER stress pathways in the SC alleviates pain (197, 198). ER stress in DH neurons can influence the environment and promote spinal sensitization and increased pain sensitivity (199). ROS production is an outcome of ER stress. GABAergic interneurons are sensitive to ROS. The increased production of ROS following ER stress causes long-term depression of GABAergic interneurons in the DH and impairs GABA release following SNL (197, 200). It is possible that similar mechanisms occur in EAE. In addition, astrocytes and macrophages release pro-nociceptive cytokines including IL-1β and IL-6 in response to ER stress (201, 202). These cytokines are upregulated in MS/EAE and they have been implicated in pain (130, 203). X-Box Binding Protein 1 (XBP1), a transcription factor associated with ER stress and a regulator of unfolded protein response, has been implicated in neuropathic pain (199, 204). XBP1 increases in the brain white matter in MS and in SC white matter in EAE. However, the potential contribution of XBP1 to neuropathic pain in MS/EAE has not received attention. In sum, the involvement of ER stress in pain responses in EAE or MS and its relation to BK is an intriguing research direction that warrants further studies.

### Ion Pumps and Exchangers

Plasma membrane calcium ATPases (PMCAs) and NCX are among ion pumps and exchangers, respectively, that are modulated during MS/EAE, and implicated in pain mechanisms. Their main physiological function is to regulate intracellular Ca^2+^ concentrations. Both PMCA and NCX expel intracellular Ca^2+^. PMCAs are essential in maintaining the cytosolic Ca^2+^ concentrations due to their high Ca^2+^ affinity and low Ca^2+^ capacity, whereas NCX, which has low Ca^2+^ affinity, participates more dynamically in re-establishing Ca^2+^ homeostasis during large cytosolic Ca^2+^increases (205). Aberrant Ca^2+^ clearance in neurons can cause hyperactivity (206).

Studies on MOG_35−55_-induced chronic EAE have shown that the levels of one of the PMCA isoforms, PMCA2, which is exclusively expressed in neurons, is downregulated in the DH of mice at onset of the disease. This is paralleled by a concomitant increase in mechanical and thermal pain sensitivity (207). PMCA2 is also downregulated in motor neurons (208, 209) and photoreceptors at early stages of EAE, causing aberrance in Ca^2+^ signaling and contributing to synaptic dysfunction (210). Since PMCA2 is not expressed in the DRG (211), it is likely that the modulation of pain responses by PMCA2 is primarily mediated through changes in DH neurons. It has been postulated that a delay in Ca^2+^ clearance, as a result of decreased PMCA2 in DH neurons of EAE mice, could lead to increased intracellular Ca^2+^ resulting in the hyperactivation of DH neurons. Furthermore, increased intracellular Ca^2+^ could activate Ca^2+^-dependent transcription factors involved in the transcription of pro-nociceptive genes (207). It is worth noting that glutamate, which is one of the major players in the initiation and maintenance of chronic pain (138, 212, 213) and central sensitization (33), modulates PMCA2 levels in SC neurons. Kainic acid, an analog of glutamate, reduces PMCA2 levels in cultured SC neurons and administration of 2,3-dioxo-6-nitro-7-sulfamoyl-benzo quinoxaline (NBQX), an AMPA/Kainate receptor antagonist, restores PMCA2 levels in EAE mice (148). IL-1β is also a trigger that reduces PMCA2 expression by mechanisms that remain elusive (207). Further studies are needed to establish the direct link between PMCA2 and pain mechanisms in DH neurons.

### NCX

NCX has been studied in animal models of neuropathic pain (214). Because of its intrinsic properties as an exchanger, this transporter allows the passage of Ca^2+^ and Na^+^ across the membrane in either direction, depending on ion gradients. In the forward mode, NCX mediates intracellular Ca^2+^ extrusion whereas in the reverse mode it facilitates Ca^2+^ influx. Reverse activity of NCX has been reported in animal models of neuropathic pain and EAE (215, 216). When the reversal of NCX activity is inhibited by pharmacological agents in rodents with peripheral nerve injury, the Ca^2+^ overload in the lumbar DRG is reduced and an overall decrease in pain sensitivity is observed (214).

During EAE, the number of NCX expressing neurons in the lumbar SC increases. NCX co-localizes with Nav1.6 channels in the dorsal column white matter (216). An increase in the number of Na^+^ channels and the consequent elevation in Na^+^ currents, reverses NCX activity leading to Ca^2+^ overload and axonal injury in the CNS during EAE (216, 217). The co-localization of Nav1.6 and NCX is also observed in cervical SC tissue of MS patients (218). NCX is expressed in cortical astrocytes where it facilitates glutamate release (219). Reactive astrocytes participate in the modulation of neuropathic pain by releasing glutamate, among other modulators of pain (220). Taken together, these investigations raise the possibility that NCX contributes, not only to axonal damage, but also to mechanisms underlying pain hypersensitivity by modulating Ca^2+^ signaling. This mechanism has not been adequately explored in EAE-related pain, and could be an engaging research direction.

The involvement of ion channel and exchangers in pain mechanisms has been summarized in Table 1.

**Table 1:** Summary of studies implicating ion channels in pain and their principal findings.

**Channel**	**Experimental paradigm**	**Outcomes**	**References**
CaV2.2	CFA-induced inflammation	• Upregulation of CaV2.2 expression • Increased voltage in CaV2.2 currents and frequency of action potentials • Thermal hyperalgesia	(167)
	CaV2.2 knockout	Decreased neuropathic and inflammatory pain	(168)
	Interference with CaV2.2 trafficking in DRG neurons	• Downregulation of pro-nociceptive neurotransmitter release • Reduction of excitatory synaptic transmission in DH neurons • Attenuation of inflammatory and neuropathic pain	(170)
	CCI	Upregulation of CaV2.2 in DH neurons	(171)
	SNL	Downregulation of CaV2.2 in corresponding, injured DRG and upregulation in adjacent uninjured DRG	(172)
	Inhibition in DRG neuronal cultures	Reduced pro-nociceptive neurotransmitter release	(175)
	Nerve injury	Synaptic targeting of NMDA-R	(176)
	Models of neuropathic pain	Ectopic neurite sprouting	(177)
Nav 1.6	SNL; knockdown in DRG neurons	• Reduced frequency of action potentials and hyperexcitability • Reduced pain	(189)
	Spared nerve injury; knockdown in DRG	• Reduced DRG excitability • Improved pain sensitivity	(190)
Nav1.8	CFA-induced pain	• Upregulation of expression in DRG neurons • Increased excitability of DRG neurons	(160)
K^+^	EAE/MS	• Reduced b4 subunit of BK channels • Hyperexcitability of DRG neurons	(194)

## Additional Contributors to Pain Mechanisms in EAE: Transcription Factors, Signaling Pathways and Inflammatory Mediators, E.G., Chemokines

Several molecular mechanisms have been implicated in the pathogenesis and maintenance of neuropathic pain in MS/EAE. Most of these studies were undertaken to investigate molecules that been implicated at different stages of disease pathophysiology.

### Wnt Signaling Pathways

The Wnt signaling pathways are involved in the development of EAE-associated chronic pain by increasing the expression of pro-inflammatory and pro-nociceptive cytokines (221). The physiological roles of Wnt include CNS development (222), synaptic plasticity (223), regulation of oligodendrocyte maturation and differentiation (224) and cytokine production by cortical and SC neurons (225, 226). These functions are exerted through β-catenin-dependent and -independent pathways (227, 228). During EAE, both of these pathways are overactivated, and pharmacological inhibition of either of these pathways attenuates mechanical allodynia (221). Similar results were obtained by use of inflammatory and neuropathic pain models, supporting further the involvement of Wnt in pain mechanisms. Wnt signaling enhances synaptic plasticity and spine morphogenesis in DH neurons. This was associated with hypersensitization and chronic pain in adult mice (229).

### C-X3-C Motif Chemokine Ligand 1 (CX3CL1) and CX3C Receptor 1(CX3CR1)

Chemokines are potent mediators of inflammation in MS, with T-cells being the major source during the initial phase of the disease. They chemoattract leukocytes to affected sites (230). An increase in chemokines and their receptors is observed in the blood and CSF of individuals with MS (231) as well as animals affected by EAE (232).

The chemokine CX3CL1 and its receptor CX3CR1 have been implicated in pain mechanisms. In the CNS, CX3CL1 is primarily expressed in neurons, and CX3CR1, is predominantly found in microglia. The involvement of CX3CR1 in pain mechanisms is indicated by the fact that CX3CR1 knockout mice do not develop thermal hyperalgesia in an inflammatory pain model (233). Moreover, upon interaction with its ligand in the DH, CX3CR1, which is a G-coupled receptor (234), initiates a cascade of events in microglia, with ultimate production and release of pro-nociceptive mediators such as IL-1β, IL-6 and NO (235).

The link between pain and CX3CL1 was further demonstrated in a R-R EAE model. The authors showed that induction of pain by ligation of the middle, sensory branch of the trigeminal nerve during a remission, increases CX3CL1 expression in the lumbar SC leading to the recruitment of immune cells which eventually results in EAE relapse (236). Another study found that CX3CL1 and CX3CR1 are upregulated in the DRG and DH of EAE rats at the early stage of the disease, prior to demyelination and axonal injury (126). This corresponded with the onset of hypoalgesia (126). The authors postulated that the parallelism between hypoalgesia, a sensory aberrance manifesting prior to the development of hyperalgesia in MS (237), and the increase in CX3CL1 and CX3CR1 in the DRG and DH support the idea that they play a role in nociception and pain.

### BDNF, Tyrosine Receptor Kinase B (TrkB) and Extracellular Signal-Regulated Kinase (ERK) Signaling Pathway

BDNF has been studied in the context of MS/EAE because of its involvement in MS-associated neuroinflammation (238), and the possibility of using it as a marker for disease progression (239), but its role in chronic pain has been controversial. A variety of animal models have shown both anti-nociceptive and pro-nociceptive effects of BDNF. Interestingly, ERK and c-fos, which are among the downstream signaling molecules activated by BDNF and its receptor TrkB, are considered markers of central sensitization (240). Phospho ERK (pERK) directly increases the activity of AMPA and NMDA receptors while decreasing the activity of K^+^ channels in the lumbar DH. The overall result is an increase in the excitability of superficial laminae neurons (241). These rapid events associated with pERK activation are followed by the slower but long-lasting effects such as the transcription of genes implicated in pain including c-fos, Cox-2 and TrkB. Thus, pERK plays a critical role in central sensitization of DH neurons (241).

In the DH of EAE mice, cellular markers of central sensitization, including pERK, are increased in neurons, and this is paralleled by mechanical and cold hyperalgesia (31). In R-R EAE, attenuation of BDNF-TrkB-ERK signaling in the lumbar DH, is associated with the alleviation of mechanical allodynia in the diseased mice (242). Since white blood cells of individuals with R-R MS express elevated BDNF levels (243), it is likely that immune cells infiltrating the CNS are a source of BDNF in addition to glia and neurons. In rats with EAE, BDNF in the DRG and SC was upregulated at the peak of the disease (244). In agreement, TrkB levels were elevated in the lumbar DH and associated with increased mechanical pain sensitivity in R-R EAE (242).

### IL-1β – NF-kB Signaling Pathway

In the context of neuropathic pain, IL-1β, IL-6 and TNFα have been implicated in the maintenance of pain states following injury and disease (131, 245, 246). These mediators exert their actions in a synergistic manner. Upon binding to their respective receptors, they can lead to the production, secretion and modulation of additional cytokines (131). Both activated glia and infiltrating immune cells release IL-1β, IL-6 and TNFα (247, 248).

IL-1β is produced in an inactive precursor form. Cleavage of the precursor by caspase-1 produces mature IL-1β which is the active form. The binding of IL-1β to its receptor induces the activation of transcription factors such as NF-kB and this results in the further production and release of pro-inflammatory molecules including other cytokines, ROS, NO, NOS and cyclooxygenases (COXs) (249, 250). It has been proposed that in rodents experiencing inflammation-evoked hyperalgesia, IL-1β activates NF-kB in DRG and SC neurons inducing COX-2 (251). COX-2 is a potent stimulator of prostaglandins, which are lipids that exert several physiological functions including maintenance of inflammation and pain (252, 253). IL-1β also enhances mechanical and thermal pain sensitivity by perturbating the activity of glutamate receptors (254–256). IL-1 receptor I, which is expressed in nociceptors (257), colocalizes with the NR1 subunit, one of the NMDA-R subunits (246). IL-1β selectively induces the phosphorylation of NR1 subunit facilitating pain transmission (248). Furthermore, administration of IL-1 receptor antagonist (IL-1ra) significantly reduces inflammatory hyperalgesia in rats, by inhibiting NR1 phosphorylation in the SC (255).

IL-1β, IL-6 and TNFα have been investigated in MS pathology and related pain (258, 259). As stated before, the majority of the intracellular signaling pathways that lead to neuropathic pain during MS/EAE include the transcription of pro-inflammatory and pro-nociceptive cytokines such as TNFα, IL-6 and IL-1β. IL-1β is necessary for the neuroinflammatory reaction that develops during EAE as indicated by the resistance of IL-1β knockout mice to EAE (260). IL-1β has been associated with pain hypersensitivity in mice with EAE, as systemic administration of an IL-1 receptor antagonist alleviates pain (203).

**Figure 1:**
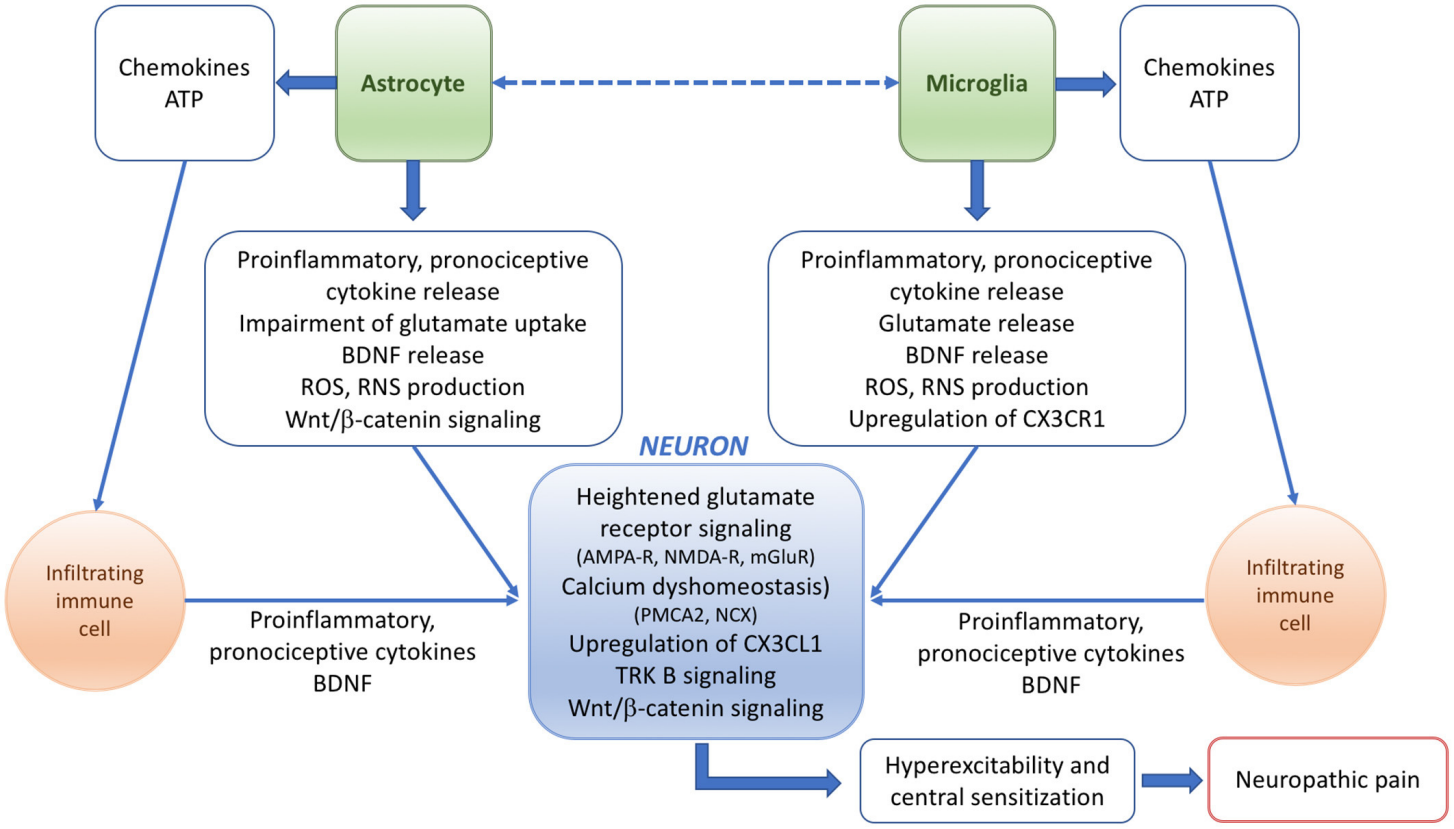
Scheme summarizing the potential mechanisms and players underlying the pathophysiology of neuropathic pain in EAE or MS.

The molecular mechanisms underlying pain responses in pathological conditions including EAE have been summarized in Figure 1.

## Conclusions

In spite of a wealth of knowledge about the immune system reaction and the crosstalk between the immune and nervous systems in MS and EAE, information regarding the mechanisms underlying neuropathic pain has been limited. Given the high frequency of pain states in individuals affected by MS, it is imperative to advance the understanding of events leading to the onset and maintenance of chronic pain. Revealing the specific aberrant mechanisms and major players involved in the development and/or maintenance of pain in MS/EAE would greatly alter the pharmacological approach to its treatment, thus improving the quality of everyday life in affected individuals. Considering the type of pain experienced in MS, it is highly likely that diverse and complex mechanisms regulate pain processing. In particular, neuropathic pain remains a challenge, not only in MS but in other pathological conditions of the CNS. Whereas such mechanisms could be somehow distinct in each pathological condition, commonalties also exist. Because neuropathic pain has been extensively studied in animal models of nerve injury, findings obtained in such studies could facilitate future investigations on MS/EAE-associated pain. For example, glutamate excitotoxicity-induced death of inhibitory GABAergic neurons in the superficial DH has been implicated in the transition of acute neuropathic pain into chronic neuropathic pain following nerve injury (261). As mentioned above, a role for glutamate excitotoxicity has been shown in EAE and high glutamate levels have been reported in the CSF of individuals with MS. Therefore, mechanisms similar to those reported in nerve injury (261) could also underlie chronic neuropathic pain in EAE/MS.

Studies on EAE have also shown that demyelination and inflammation occur, not only in the CNS, but also in the PNS (262). Peripheral nerve demyelination has been reported in a subset of individuals with MS (263). This can affect the function of sensory neurons in the DRG. In fact, electrophysiological studies indicated membrane hyperexcitability of DRG sensory neurons and therefore, peripheral sensitization in EAE (195, 264).

Regarding the use of EAE for the study of pain states in MS, the multitude of EAE models that have been used in investigations, could challenge the acquisition of cohesive information (265). On the other hand, corroborating findings in more than one model is needed to determine the wider applicability of the findings to different species and experimental paradigms.

With respect to the management of chronic pain, the challenge experienced in other pathological conditions associated with neuropathic pain, are also relevant to MS. Both pharmacological and non-pharmacological approaches are used, although further studies are needed to establish the effectiveness of these therapies (69, 266, 267). Importantly, the evaluation of pharmacological therapies in EAE or other models of MS, need to be performed in a manner that can be applicable in the clinic.

## Author Contributions

EM and SE conceived and wrote this article and approved the submitted version.

## Funding

The research in the authors' laboratory has been supported by the Reynolds Family Spine Laboratory Funds and the New Jersey Commission on Spinal Cord Research (Grant CSCR17IRG007 to SE).

## Conflict of Interest

The authors declare that the research was conducted in the absence of any commercial or financial relationships that could be construed as a potential conflict of interest.

## Publisher's Note

All claims expressed in this article are solely those of the authors and do not necessarily represent those of their affiliated organizations, or those of the publisher, the editors and the reviewers. Any product that may be evaluated in this article, or claim that may be made by its manufacturer, is not guaranteed or endorsed by the publisher.
